# Circulating Complement-C1q TNF-Related Protein 1 Levels Are Increased in Patients with Type 2 Diabetes and Are Associated with Insulin Sensitivity in Chinese Subjects

**DOI:** 10.1371/journal.pone.0094478

**Published:** 2014-05-14

**Authors:** Xuebo Pan, Tingting Lu, Fan Wu, Leigang Jin, Yi Zhang, Lihua Shi, Xiaokun Li, Zhuofeng Lin

**Affiliations:** 1 School of Pharmacy, Wenzhou Medical University, Wenzhou, China; 2 Engineering Research Center of Bioreactor and Pharmaceutical Development, Jilin Agricultural University, Changchun, China; University of Hong Kong, China

## Abstract

**Background:**

Complement-C1q TNF-related protein 1 (CTRP1), a member of the CTRP superfamily, possesses anti-inflammatory and anti-diabetic effects in mice. However, the clinical relevance of CTRP1 has been seldom explored. The current study aimed to investigate the association of circulating CTRP1 and type 2 diabetes mellitus (T2DM) in a Chinese population.

**Design and Methods:**

Serum CTRP1 and adiponectin levels of 96 T2DM patients and 85 healthy subjects were determined by ELISA, and their associations with adiposity, glucose and lipid profiles were studied. In a subgroup of this study, the 75-g oral glucose tolerance test (OGTT) was performed in 20 healthy and 20 T2DM subjects to evaluate the relationship among serum levels of CTRP1 and adiponectin, insulin secretion and insulin sensitivity.

**Results:**

Serum CTRP1 levels were significantly increased in patients with T2DM, compared with healthy controls (p<0.001). Similar to adiponectin, serum levels of CTRP1 were significantly correlated to several parameters involved in glucose metabolism and insulin resistance, and independently associated with fasting glucose levels (p<0.05) after BMI and gender adjustments. Furthermore, CTRP1 levels were positively correlated to insulin secretion, while negatively to insulin sensitivity, as measured by OGTT.

**Conclusion:**

CTRP1 is a novel adipokine associated with T2DM in humans. The paradoxical increase of serum CTRP1 levels in T2DM subjects may be due to a compensatory response to the adverse glucose and lipid metabolism, which warrants further investigation.

## Introduction

Adiponectin is a member of the C1q/TNF-related protein (CTRP) family predominantly expressed in adipose tissue. Animal-based studies have shown that adiponectin has anti-inflammatory and insulin-sensitizing properties [1], [2], [3], [4]. Clinical studies suggest a potential correlation between adiponectin and the onset of diabetes; elevated concentrations of adiponectin are strongly and independently associated with reduced risk of incident type 2 diabetes mellitus (T2DM) in apparently healthy individuals [5], whereas lowered circulating levels of adiponectin (<4 µg/mL) are closely associated with increased susceptibility to T2DM [6].

In addition to adiponectin, other 15 CTRP family members have been identified [7], resembling adiponectin in regulating glucose and energy metabolism [8], [9], [10], [11]. As a member of CTRP family, CTRP1 is mainly expressed in adipose tissue [12], and derived mostly from stromal vascular cells (SVC) composed of adipose-tissue macrophages, preadipocytes and endothelial cells [9], [12]. Applying recombinant CTRP1 activates the serine/threonine protein kinase Akt and mitogen-activated protein kinase (MAPK) signaling pathways in mouse myotubes *in vitro* [9]. Importantly, endogenous administration of recombinant CTRP1 significantly reduces serum glucose levels in mice [9]. Furthermore, in isolated soleus muscle, recombinant CTRP1 can activate the AMP-activated protein kinase (AMPK) signaling pathway to increase fatty acid oxidation [13]. Notably, CTRP1-transgenic mice display the dramatic enhancements of insulin sensitivity, fatty acid oxidation and energy expenditure, in contrast to the significant decrease of high-fat diet-induced weight gains [13]. Based on the above-mentioned observations, CTRP1 seems to play an important role in the regulation of lipid metabolism, which provides the entry point for its potential use in therapy.

Despite the numerous animal-based studies suggesting CTRP1 as a metabolic regulator with multiple beneficial effects on obesity and diabetes, the clinical relevance of CTRP1 has been little explored. Recent human studies have indicated that the circulating levels of CTRP1 were significantly increased in subjects with hypertension [14] and metabolic syndrome [15]. However, no data has been available concerning the relationship between CTRP1 and T2DM.

To explore the physiological and pathological role of CTRP1 in T2DM, as well as its relation with adiponectin, we measured the serum concentrations of CTRP1 and adiponectin in 181 Chinese subjects, and analyzed its associations with a cluster of metabolic parameters. The 75-g oral glucose tolerance test (OGTT) was further performed to explore the association between CTRP1 and insulin secretion as well as insulin sensitivity.

## Materials and Methods

### Subjects

Ninety-six patients with T2DM and eighty-five age- and sex-matched healthy controls (n = 85) were recruited from Wenzhou Diabetes Center. Diabetes was diagnosed according to the criteria suggested by the American Diabetic Association (1997). All subjects in T2DM group received no treatment before recruitment, and those with the following conditions were excluded from this study: biliary obstructive diseases, acute or chronic virus hepatitis, cirrhosis, known hyperthyroidism or hypothyroidism, presence of cancer, current treatment with systemic corticosteroids and pregnancy. Current drinkers and ex-drinkers, as defined in our previous report [16], were also excluded. The present study was approved by the Human Research Ethics Committee of Wenzhou Medical University, and the written informed consents were obtained from all the participants.

### Anthropometric and Biochemical Measurements

All the subjects received comprehensive physical examinations and routine biochemical analyses of blood. Body mass index (BMI) was calculated as weight/height^2^ (kg/m^2^). Waist circumference was measured midway between the lower rib margin and the iliac crest on the midaxillary line. Resting blood pressure (BP) was measured using an electronic sphygmomanometer (Tensoval duo control, Germany) according to the standardized protocol.

Blood samples were collected after a 12-hour overnight fast for measurement of glucose, triglyceride, total cholesterol, LDL-cholesterol and HDL-cholesterol using the Hitachi 7170 Analyzer (Boehringer Mannheim, Mannheim, Germany). Serum creatinine was measured by the picric acid method. HbA1c was measured by automated liquid chromatography (Bio-Rad VARIANT II Hemoglobin Testing System, Hercules, CA) with the normal range 4.1–6.1%.

### Insulin Measurement and Calculation

A 75-g oral glucose tolerance test (OGTT) was performed on 20 patients with T2DM and 20 healthy controls. Circulating levels of insulin were measured by radioimmunoassay (Linco Research Inc., St Charles, MO), and C-peptide were quantified using a chemiluminescence immunoassay on the Bayer 180SE Automated Chemiluminescence Systems (BayerAG Leverkusen, Germany). The homeostasis model assessment of insulin resistance (HOMA-IR) was calculated using the following equation: fasting serum insulin concentration [FINS] (mU/l) * fasting plasma glucose [FPG] (mmol/l)/22.5 [17]. Basal insulin secretion index (HOMA-%B) was calculated as (FINS [mU/l] * 6−3.33)/(FPG [mmol/l]−3.5) [18]. Insulin sensitivity index in OGTT was calculated according to Cederholm formula (ISI-Cederholm) [19]. Insulin action index (IAI) were calculated as (−Ln[FPG×FINS]) [20].

### Measurement of Serum Levels of Adiponectin and CTRP1

Serum samples were collected during 9∶00 am to 11∶00 am. Levels of adiponectin and CTRP1 were measured with the commercial ELISA kits manufactured by Antibody and Immunoassay Services (the University of Hong Kong, Hong Kong) [21] and Biovendor (Czech Republic) [15], respectively. The CTRP1 assay was highly specific to human CTRP1 without cross-reacting with other family members, and the intra- and inter assay variations were 2.7% and 8.5%, respectively.

### Statistical Methods

All statistical calculations were performed with SPSS 11.0 (SPSS, Inc., Chicago, IL). Normally distributed data were expressed as mean ± SD. Data that were not normally distributed, as determined using Kolmogorox-Smirnov test, were logarithmically transformed before analysis and expressed as median with interquartile range. χ^2^-test and one-way ANOVA were used for comparisons of categorical and continuous variables respectively. Pearson's correlation analyses were used to examine the relationship between serum CTRP1 and other parameters. To determine the parameters independently associated with CTRP1 levels, parameters including age, gender and significant correlates with CTRP1 in bivariate analyses were tested by stepwise linear regression analysis. Two-sided values of P<0.05 were considered significant.

## Results

### Characteristics of Study Subjects

Characteristics of subjects with T2DM (n = 96) and the corresponding age- and sex-matched controls (n = 85) were described in Table 1. Compared with healthy controls, the diabetic patients had greater values of BMI, waist circumference, systolic blood pressure (SBP), diastolic blood pressure (DBP), FPG, FIN, HOMA-IR, HbAc1(%), LDL-cholesterol, HDL-cholesterol and C-reactive protein (Hs-CRP) (p<0.05 for all parameters).

**Table 1:** Anthropometric, clinical and biochemical characteristics of T2DM and healthy control groups.

Variables	Healthy (n = 85)	T2DM (n = 96)	P value
Age (years)	45.25±1.94	46.13±2.42	NS
Male sex (%)	43 (51)	47 (49)	-
BMI	21.76±0.42	24.51±0.90	<0.001
Waist circumference (cm)	60.5±10.6	86.71±8.52	0.027
SBP (mmHg)	107.9±3.30	127.10±5.96	<0.001
DBP (mmHg)	75.30±1.90	81.31±2.50	0.023
Fasting glucose (mmol/l)	5.07±0.11	9.35±0.63	<0.001
Fasting insulin (mIU/l)	6.75±0.43	12.46±1.77	<0.001
Fasting C-peptide (nmol/l)	1.81±0.13	3.21±0.25	<0.001
HOMA-IR	1.92±0.24	4.24±0.70	0.005
Insulin action indexes	−3.57±0.12	−4.27±0.17	0.003
HbA_1c_, % (mmol/mol)	5.44±0.08	8.48±0.65	<0.001
Triglyceride (mmol/l)	1.49±0.17	1.50±0.15	NS
Total cholesterol (mmol/l)	4.85±0.16	5.27±0.38	NS
HDL-chlosterol (mmol/l)	1.33±0.05	1.25±0.08	0.020
LDL-chlosterol (mmol/l)	2.60±0.15	2.40±0.28	NS
C reactive protein (µg/ml)	1.59±0.30	8.73±1.17	<0.001
Adiponectin (µg/ml) _men	14.70±1.03	5.62±0.68	<0.010
_women	17.88±2.23	5.24±0.87	<0.001
CTRP1 (ng/ml) _men	135.6±15.42	378.3±43.93	<0.001
_women	149.8±12.71	304.4±30.40	<0.001

Data are expressed as mean ± standard error of the mean. NS: not significant.

Abbreviation: BMI, body mass index; SBP, systolic blood pressure; DBP, diastolic blood pressure; HOMA-IR, homeostasis model of assessment-insulin resistance; HbA_1c_, hemoglobin *A1c;* HDL-cholesterol, high density lipoprotein-cholesterol; LDL-cholesterol, low density lipoprotein-cholesterol; *CTRP-1,* Complement-C1q TNF-related protein 1.

### Serum CTRP1 Levels were Increased in T2DM Subjects

**Figure 1:**
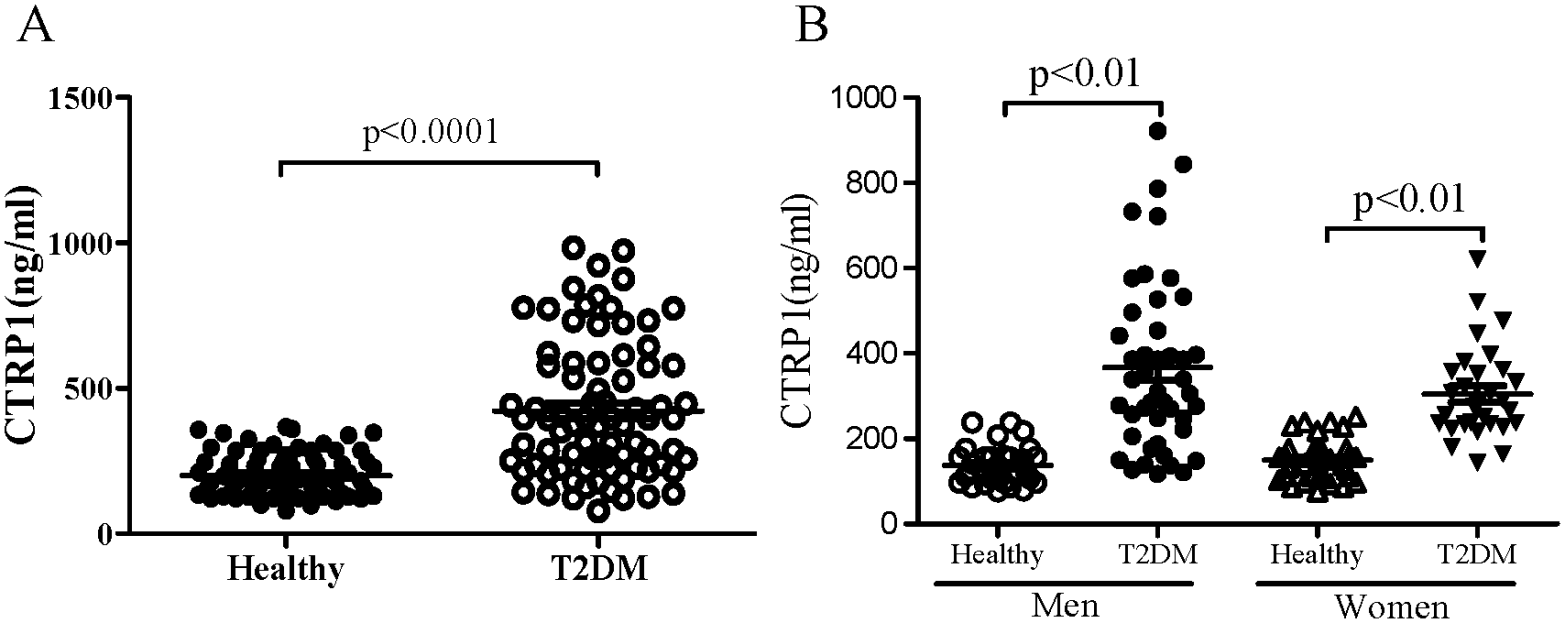
Circulating levels of adiponectin (A) and CTRP1 (B) in T2DM patients and healthy controls.

Fasting adiponectin levels ranged from 0.09 to 51.85 µg/ml among 181 subjects. Consistent with previous reports [6], [22], our results showed that, fasting serum adiponectin levels were more than 3 times lower in type 2 diabetes compared with healthy controls (5.31±0.50 µg/ml *vs.* 16.36±1.07 µg/ml; age and sex-adjusted p<0.001) (Table 1 & Figure 1A).

Fasting serum CTRP1 levels ranged from 77.7 to 1187.3 ng/ml, and the median was 233.4 ng/ml. Serum CTRP1 levels in men (358.4±4.17 ng/ml) were significantly higher than that in women (232.7±2.07 ng/ml) (p<0.05). Notably, unlike the overall change pattern of circulating adiponectin, the serum concentrations of CTRP1 were significantly higher in subjects with T2DM in both sexes than that in control subjects (378.3±43.9 ng/ml *vs.* 135.6±15.4 ng/ml in men and 304.4±30.4 ng/ml *vs.* 149.8±12.7 ng/ml in women, T2DM vs. Control; age and sex-adjusted p<0.01) (Table 1 & Figure 1B).

### Fasting CTRP1 Levels Closely Related with Glucose Metabolism

**Figure 2:**
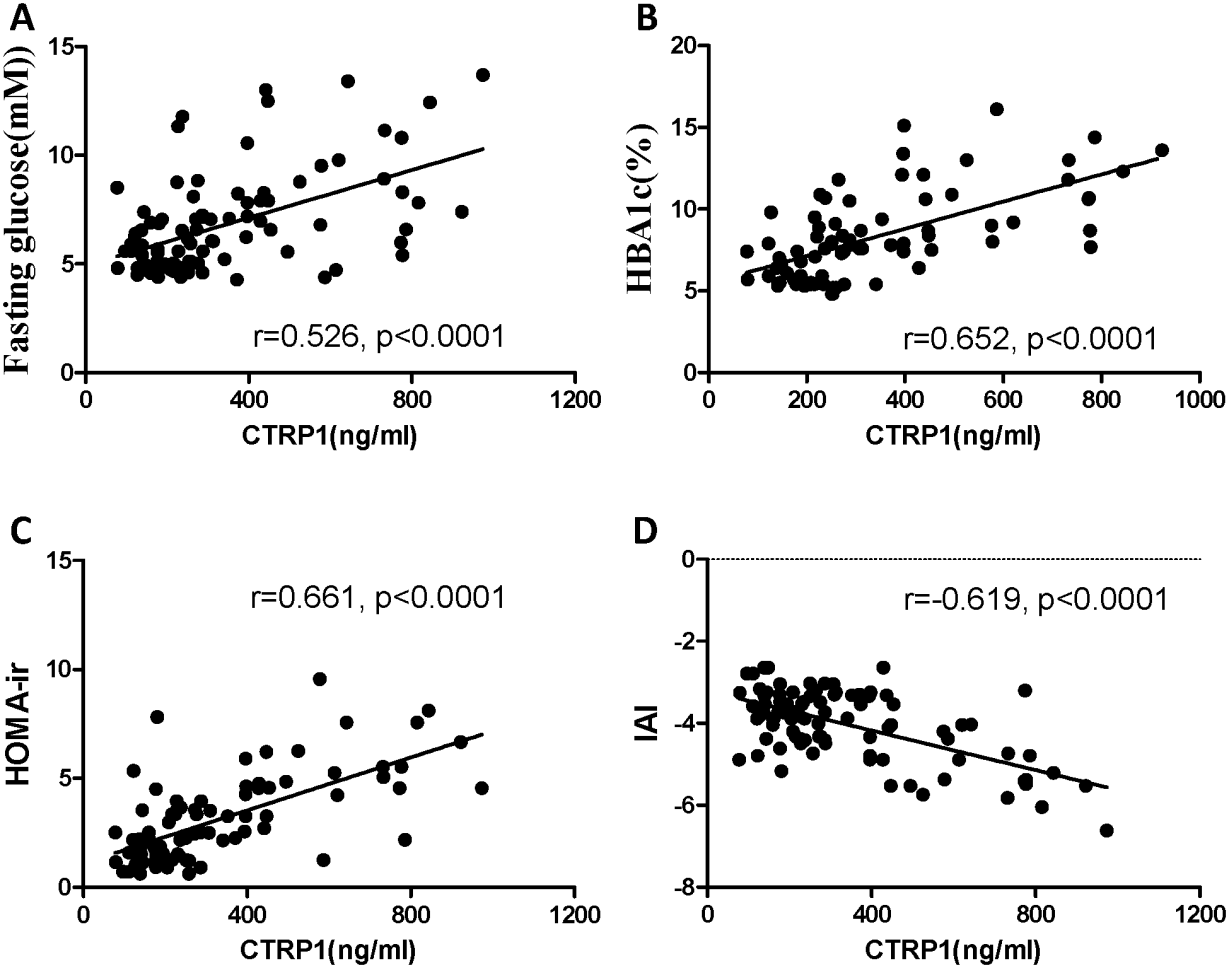
Correlation of CTRP1 with fasting glucose (A), HBA1c (B), HOMA-ir (C) and IAI (D) after gender- and BMI-adjustment in 96 T2DM subjects.

In all subjects, the levels of fasting serum CTRP1 were positively correlated with fasting glucose, 2h glucose, HbA1c, HOMA-IR, LDL-cholesterol, and negatively to those of IAI and HDL-cholesterol after adjusting for age, gender and BMI (Table 2, Figure 2, p<0.05 respectively). Notably, the levels of CTRP1 were significantly correlated to the parameters associated with glucose metabolism. As the levels of CTRP1 were different between men and women, we next analyzed the correlation separately. Interestingly, the correlation between CTRP1 and glucose metabolism was more significant in men after adjusting for age and BMI (Table 2, p<0.05 respectively).

**Table 2:** Correlations of fasting serum CTRP1 levels with relevant clinical parameters in 96 T2DM subjects.

Variables	Serum CTRP1_total subjects (adjusted by age, gender and BMI)		Serum CTRP1_male (adjusted by age and BMI)		Serum CTRP1_female (adjusted by age and BMI)	
	R	p	r	p	r	p
Fasting glucose	0.374	0.011	0.403	0.007	0.319	0.022
Fasting insulin	0.315	0.049	0.388	0.016	0.281	NS
2-h glucose	0.380	0.042	0.409	0.023	0.326	0.043
HOMA-IR	0.581	<0.001	0.611	<0.001	0.412	0.005
IAI	−0.543	0.001	−0.641	<0.001	−0.452	0.015
HbA1c	0.627	0.002	0.751	<0.001	0.301	NS
Total cholesterol	0.352	0.043	0.369	0.029	0.215	NS
HDL-cholesterol	−0.358	0.037	−0.413	0.011	−0.232	NS
LDL-cholesterol	0.391	0.013	0.319	0.042	0.435	0.001

NS: not significant. IAI, insulin action indexes.

To further investigate the relationship between CTRP1 and other anthropometric parameters, multiple stepwise regression analysis involving all the parameters including fasting glucose, fasting insulin, 2h-glucose, HOMA-IR, insulin action indexes, total cholesterol, LDL-cholesterol and HDL-cholesterol with significant correlations with serum CTRP1 was performed. Fasting CTRP1 was found to be independently associated with fasting glucose (OR 34.81 [95%CI 16.423 to 53.213]) after gender- and BMI-adjustment (p<0.05).

### Association of Serum CTRP1 with Insulin Secretion and Sensitivity through OGTT

As serum CTRP1 levels were found to strongly correlate with glucose levels, the association between serum CTRP1 levels and the variables on insulin secretion and sensitivity were further investigated through OGTT among 20 T2DM patients and 20 healthy subjects. Interestingly, serum CTRP1 levels in healthy subjects were positively correlated with FINS and HOMA-%B, as well as negatively with ISI-Cederholm (p<0.05) before and after BMI-adjustment. In T2DM subjects, CTRP1 levels were significantly positively associated with HOMA-IR, and negatively with ISI-Cederholm (p<0.05), but not associated with FINS and HOMA-%B before and after BMI-adjustment.

## Discussion

Although several animal experiments using both gain-of-function and transgenic approaches have suggested CTRP1 as an important regulator of glucose metabolism and insulin sensitivity, the clinical relevance of these findings in humans remains poorly characterized. In this study, our data showed that CTRP1 might be involved in the pathogenesis of T2DM, as supported by two novel findings. First, serum CTRP1 concentrations were significantly increased in subjects with T2DM compared with the age- and gender-matched healthy subjects, in contrast to the change pattern of circulating adiponectin levels. Second, serum CTRP1 levels were strongly associated with insulin secretion and sensitivity in both T2DM and healthy subjects.

Previous studies have indicated that CTRP family members, including adiponectin, CTRP3 and CTRP9, were involved in the regulation of glucose metabolism in mice and humans [9], [10], [11], [12]. For example, adiponectin, a member of CTRP family predominately expressed in adipose tissue, is significantly decreased in T2DM patients, as well as genetic and diet-induced murine models of obesity [3], [5], [23]. Administration of full-length or globular adiponectin ameliorates insulin resistance and hyperglycemia in diabetic mice [24]. Similarly, CTRP3 has been found to be strikingly associated with cardiac risk factors in humans [25]; CTRP9 levels are positively associated with favorable glucose or metabolic phenotypes and absence of metabolic syndrome, which are independent of serum total adiponectin concentrations [26]. Interestingly, treatment with recombinant CTRP3 or CTRP9 proteins can significantly decrease adverse glucose levels in *ob/ob* mice [10], [11]. Collectively, these data are in favor of the role that CTRP family members possibly play in glucose metabolism.

Previous studies have unveiled that adiponectin shows the gender difference during puberty, and is associated with androgen levels [27], [28]. In this study, we observed a similar discrepancy of CTRP1 levels between male and female subjects. However, what has caused this gender difference and whether this relates androgen levels are still unclear. Further studies involving a large number of participants are needed to answer these questions.

Circulating CTRP1 levels have been reported to significantly increase in subjects with hypertension [14] and metabolic syndrome [15]. In the present study, we explored the relationship between CTRP1 and T2DM. Our data showed a significant increase of circulating CTRP1 in T2DM patients compared with healthy subjects (Figure 1B), which is opposite to the decreasing trend of serum adiponectin observed in the same population, suggesting that even though both CTRP1 and adiponectin are from the same CTRP family, their clinical characteristics in diseases like T2DM may not be necessarily identical. This hypothesis is also supported by a previous study reporting that serum CTRP1 levels in adiponectin-null mice were significantly increased compared with the wild-type controls [9]. On the other hand, CTRP1, along with other paralogs, may have certain overlapping functions as adiponectin. For instance, replenishment of recombinant CTRP1 could also significantly reduce serum glucose levels in mice [9]. In sum, these data implied that the differential changes of CTRP1 and adiponectin following T2DM may exert a comparable beneficial effect in maintaining glucose homeostasis; and the paradoxical elevation of serum CTRP1 concentrations in T2DM patients might reflect a self-protective mechanism in response to abnormal glucose metabolism.

Another novel finding in the present study is the strong association between serum CTRP1 levels and insulin secretion and sensitivity in both T2DM and healthy subjects. Previous studies have demonstrated that, besides adiponectin, several members of the CTRP family, such as CTRP3 and CTRP9, are involved in the regulation of insulin sensitivity. In *ob/ob* mice, administration of recombinant CTRP3 significantly attenuates insulin-resistance [11]. CTRP9 transgenic mice are resistant to high-fat diet-induced obesity and insulin-resistance [29]. These reports suggest that some CTRP family members participate in the regulation of glucose metabolism and insulin sensitivity in mice.

Previous study has shown that, similar to adiponectin, CTRP3 and CTRP9, CTRP1 also possesses the insulin-sensitizing effect in mice. Peterson *et al.* reported that over-expression of CTRP1 significantly improved insulin-sensitivity in diet-induced obesity mice [13]. Our work, in agreement with previous studies [6], [30], showed that serum adiponectin levels were significantly associated with insulin sensitivity in subjects with T2DM. Unexpectedly, serum CTRP1 levels in T2DM patients and healthy controls were found to be negatively associated with ISI-Cederholm before and after BMI-adjustment during OGTT (Table 3), suggesting that CTRP1 is closely associated with insulin-sensitivity in humans under both pathological and physiological status. Moreover, serum CTRP1 concentrations in healthy subjects were also associated with fasting insulin levels and basal insulin secretion (HOMA-%B) (Table 3), suggesting that serum CTRP1 levels are related to insulin secretion in human in physiological conditions.

**Table 3:** Correlations of serum CRTP1 levels with insulin secretion and sensitivity indices in 20 T2DM and 20 healthy subjects by OGTT.

	Healthy		T2DM		Total	
	r	p	r	p	r	p
Fasting insulin levels	0.587	0.014	0.285	NS	0.392	0.014
HOMA-%B	0.550	0.022	−0.285	NS	−0.226	NS
HOMA-IR	0.365	NS	0.581	0.007	0.638	<0.001
ISI-Cederholm	−0.516	0.034	−0.522	0.019	−0.540	<0.001

Abbreviation: HOMA-%B, basal insulin secretion; ISI-Cederholm, insulin sensitivity index in OGTT. NS, not significant.

Finally, our present study also unveiled a close association between serum CTRP1 levels and adverse lipid profiles in T2DM patients. Prior literatures showed that, of all CTRP paralogs, several members including adiponectin, CTRP3 and CTRP15 were found to get involved in the regulation of lipid metabolism in mice [3], [8]. However, despite the improvement of fatty acid oxidation and energy expenditure in CTRP1 transgenic mice, blood lipid profiles, including serum triglyceride, total cholesterol, HDL-cholesterol, LDL-cholesterol, and liver and muscle triglycerides were comparable in CTRP1 transgenic and WT mice [13]. Interestingly, our present data indicated that serum CTRP1 levels were closely associated with HDL-cholesterol and LDL-cholesterol in T2DM patients, suggesting that the association between CTRP1 and other indices under the T2DM condition may be species-specific.

There are several limitations of this study. For example, the sample size was relatively small. The correlation between CTRP1 and insulin sensitivity or dyslipidemia did not guarantee the existence of the causal relationship. Further prospective studies are wanted to determine whether elevated serum CTRP1 is the enabling step of diabetes, obesity and dyslipidemia, or simply an accompanying or secondary response to these diseases.

In summary, this study demonstrated that, unlike the decrease of serum adiponectin levels, serum CTRP1 levels were significantly increased in patients with T2DM, and closely associated with insulin secretion and sensitivity in both T2DM and healthy subjects. Thus, CTRP1 is a possible regulator of systemic glucose metabolism in humans. The detailed mechanism mediating the increase of serum CTRP1 levels in T2DM patients requires further investigation.
